# Accelerating rare disease detection: an experience of multidisciplinary team model in undiagnosed diseases program in a children’s hospital

**DOI:** 10.3389/fpubh.2024.1373649

**Published:** 2024-09-17

**Authors:** Yu Shi, Shijian Miao, Yuan Yuan, Yang Fu, Chengjun Sun, Hongsheng Wang, MengMeng Ge, Dongyun Li, Guomei Shen, Xuan Gao, Xiaowen Zhai

**Affiliations:** ^1^Outpatient and Emergency Management Office, National Children’s Medical Center/Children’s Hospital, Fudan University, Shanghai, China; ^2^Department of Gastroenterology, National Children’s Medical Center/Children’s Hospital, Fudan University, Shanghai, China; ^3^Department of Respiratory, National Children’s Medical Center/Children’s Hospital, Fudan University, Shanghai, China; ^4^Department of Hematology, National Children’s Medical Center/Children’s Hospital, Fudan University, Shanghai, China; ^5^Department of Endocrinology and Inherited Metabolic Diseases, National Children’s Medical Center/Children’s Hospital, Fudan University, Shanghai, China; ^6^Department of Neonatology, National Children’s Medical Center/Children’s Hospital, Fudan University, Shanghai, China; ^7^Department of Child Health Care, National Children’s Medical Center/Children’s Hospital, Fudan University, Shanghai, China

**Keywords:** undiagnosed diseases, undiagnosed disease program, multidisciplinary, China, children

## Abstract

**Background:**

A definite diagnosis goes undiscovered for a percentage of children with undiagnosed disorders, with significant medical, psychological, and social effects. Other than specialized clinical centers, exceptional molecular studies, common procedures, and devoted activities at the national and international levels, children with complex undiagnosed disorders require innovative approaches.

**Methods:**

In March 2016, Children’s hospital of Fudan university represented the Children’s Undiagnosed Diseases Program (UDP). The purpose of this study is to describe the project findings and underline the critical significance of multidisciplinary teamwork in China’s undiagnosed rare illnesses program. We investigated the 758 cases in our UDP system retrospectively. Demographic information, laboratory test results, and genetic information were gathered.

**Results:**

Between January 2017 and December 2021, 758 cases were examined. Males made up 436 (57.5%) of the total. Over half of the patients were children under the age of five. The average patient course time preceding admission to UDP was 6.0 months (95% CI 10.512.6). These patients visited an average of 1.8 clinics during their diagnostic journey. Except for 69 individuals (90.9%), all had more than one presenting symptom in various organs: 460 (60.7%) had neurology difficulties, 151 (19.9%) had endocrine problems, and 141 (18.6%) had immunology problems. UDP has a diagnosis rate of 61.3%. Genetic testing was performed on 469 of the 758 patients, for a genetic diagnosis rate of 15.8%. The UDP method has a sensitivity of 94.5%, a specificity of 86.4%, a positive predictive value of 92.8%, and an negative predictive value of 89.5%.

**Conclusion:**

Our UDP targets an unmet need, namely the diagnosis of patients with complicated, multisystem illnesses. Using a multidisciplinary team model approach, this UDP pilot study achieved a reasonable diagnosis success rate, increasing the possibility of more diagnoses and new scientific discoveries of difficult and rare diseases.

## Background

Undiagnosed diseases are frequently rare, sometimes extremely rare, defined as affecting fewer than five people per 10,000 in the European Union (EU) or fewer than 20,000 persons in the United States (US) (1, 2). Patients suffering from undiagnosed diseases may be on a diagnostic journey for years, even decades (3). Undiagnosed diseases constitute an unmet medical need because they contribute for a considerable fraction of the overall rare diseases burden (4). Most patients begin their diagnostic journey for their complex medical issue with a single physician but later seek additional examination through referrals to many specific subspecialists. These referrals may result in an incomplete overall assessment of the patient, and the patient will require the specialist’s teamwork to obtain a precise diagnosis. A further problem in pediatrics is that disease progression or developmentally linked changes in the patient’s physiology, many of which are growth-related, pose an extra challenge (5).

The National Institutes of Health (NIH) launched the UDP in May 2008 to address the unmet needs of individuals and families suffering from rare or multisystem disorders who remained undetected despite extensive testing. Thus far, a nationwide network of collaboration has been established, considerably benefiting a large number of these patients. The goal of NIH-UDP was to diagnose patients who remained unidentified. Furthermore, they worked to discover new illnesses and provided insight into disease mechanisms (6). Given that China’s population is many times larger than that of the US, the UDP initiative will benefit a larger number of people there. We performed the Undiagnosed Diseases Program (UDP) in March 2016 in response to the needs of pediatric undiagnosed diseases as a large national pediatric medical facility. Our UDP study looked into a multidisciplinary team concept for kids. In this section, we present our UDP’s experience and strategy, emphasizing comprehension of the benefits, limitations, and success factors of such initiatives in clinical practice.

## Methods

### The undiagnosed diseases program of children’s hospital of Fudan university

Our UDP team includes hospitalists, geneticists, radiologists, pathologists, and an access-coordinating specialist representing the majority of pediatric subspecialties. Each subspecialist brings to the evaluation experience and knowledge as both a pediatrician and a specialty physician, and they are invaluable in providing insights into the intricacies associated with specific symptoms or disease processes examined in the differential diagnosis.

The UDP inclusion criteria were as follows: undiagnosed despite evaluation by at least two specialists who assessed the patient for the objective findings. Patients who satisfied the inclusion criteria were referred to our UDP team from all around China. The UDP center registers the patient in the electronic health record and works with the family to obtain all medical records from every institution, clinic, and hospital that has cared for the patient, as well as copies of all actual imaging studies and slides of all tissue samples obtained by biopsy or tissue resection. The UDP liaison will assess if a patient satisfies the selection criteria after reading and summarizing the patient’s history. The UDP liaison is an attending pediatrician who is in charge of determining whether the patient matches the requirements, gathering medical information, and scheduling multidisciplinary expert meetings. Experts were invited by UDP center staff for consultation and debate. The 1.5–2 h team discussion always reveals fresh facets of the patient’s problems. Following the talk, patients will be scheduled for pertinent examinations and follow-up phone calls by UDP liaison and staff. This follow-up will occur every month after that. A unique file will be used to manage all patient data.

**Figure 1:**
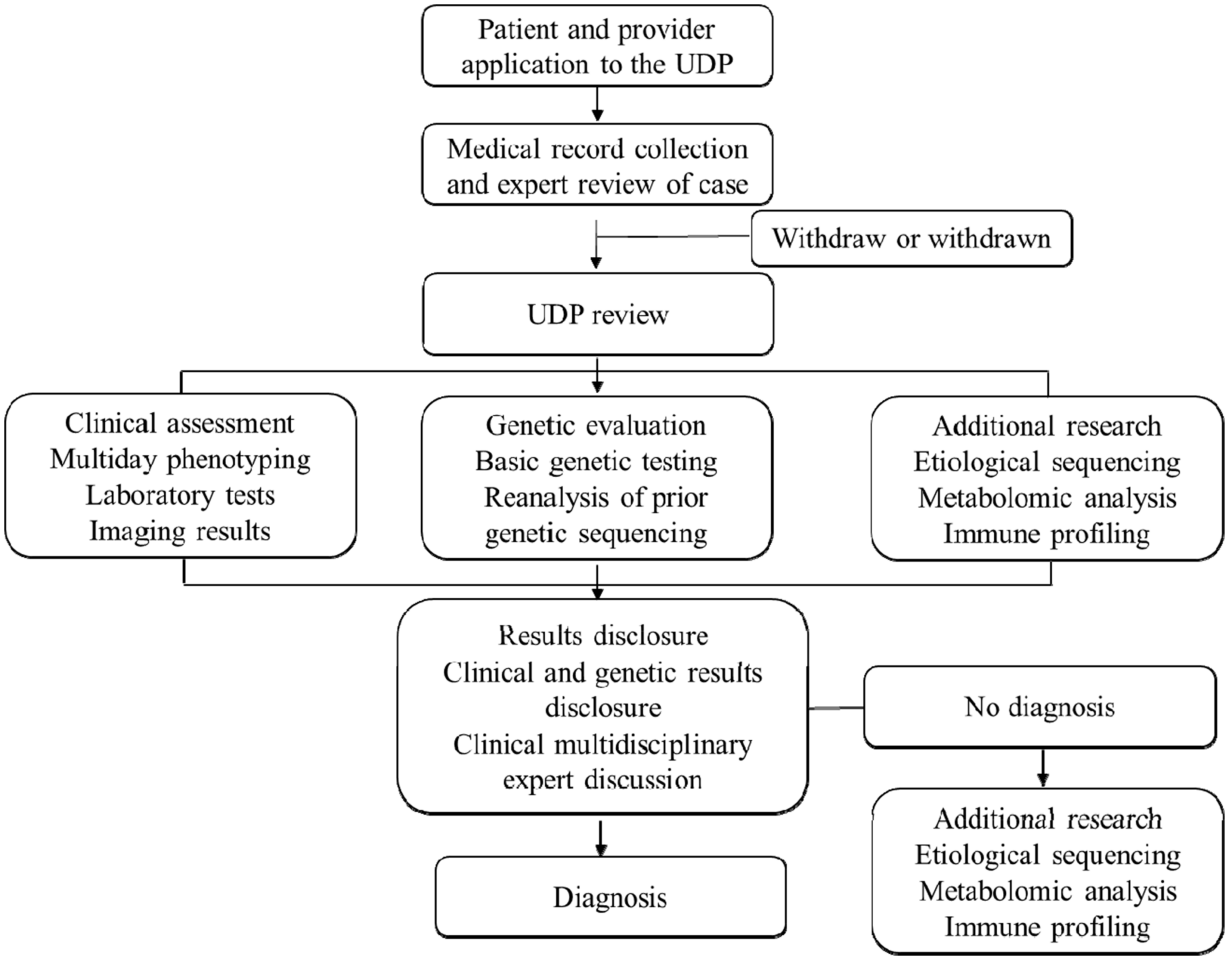
An example of the patient application and UDP participation workflow.

We employ various genetic testing methods to diagnose rare pediatric diseases, including karyotyping, fluorescence *in situ* hybridization (FISH), comparative genomic hybridization (CGH) and SNP arrays, single-gene sequencing, next-generation sequencing (NGS), targeted genomic and epigenetic analyses. Karyotyping is used to identify large-scale chromosomal anomalies, FISH detects specific DNA sequences, CGH and SNP arrays identify copy number variations, single-gene sequencing diagnoses specific diseases, NGS, including whole-genome sequencing and whole-exome sequencing, identifies rare and novel mutations, targeted genomics is used for multi-gene analysis in complex diseases, and epigenetic analysis is suitable for diagnosing disorders influenced by epigenetic factors. Each patient receives a unique genomic report, which is distributed to our clinician–scientist review panel and the patient’s physician. Sensitivity and specificity are analyzed for each genetic test by comparing test results with established diagnostic criteria or known genetic profiles. The treating physician ultimately has the last say over any new treatments recommended for the patient. Licensed genetic counselors are available to assist as needed. By incorporating detailed genetic and laboratory testing methods and sensitivity calculations, we aim to enhance the precision and reliability of diagnosing rare pediatric diseases (Figure 1).

## Results

### Patient characteristics

**Figure 2:**
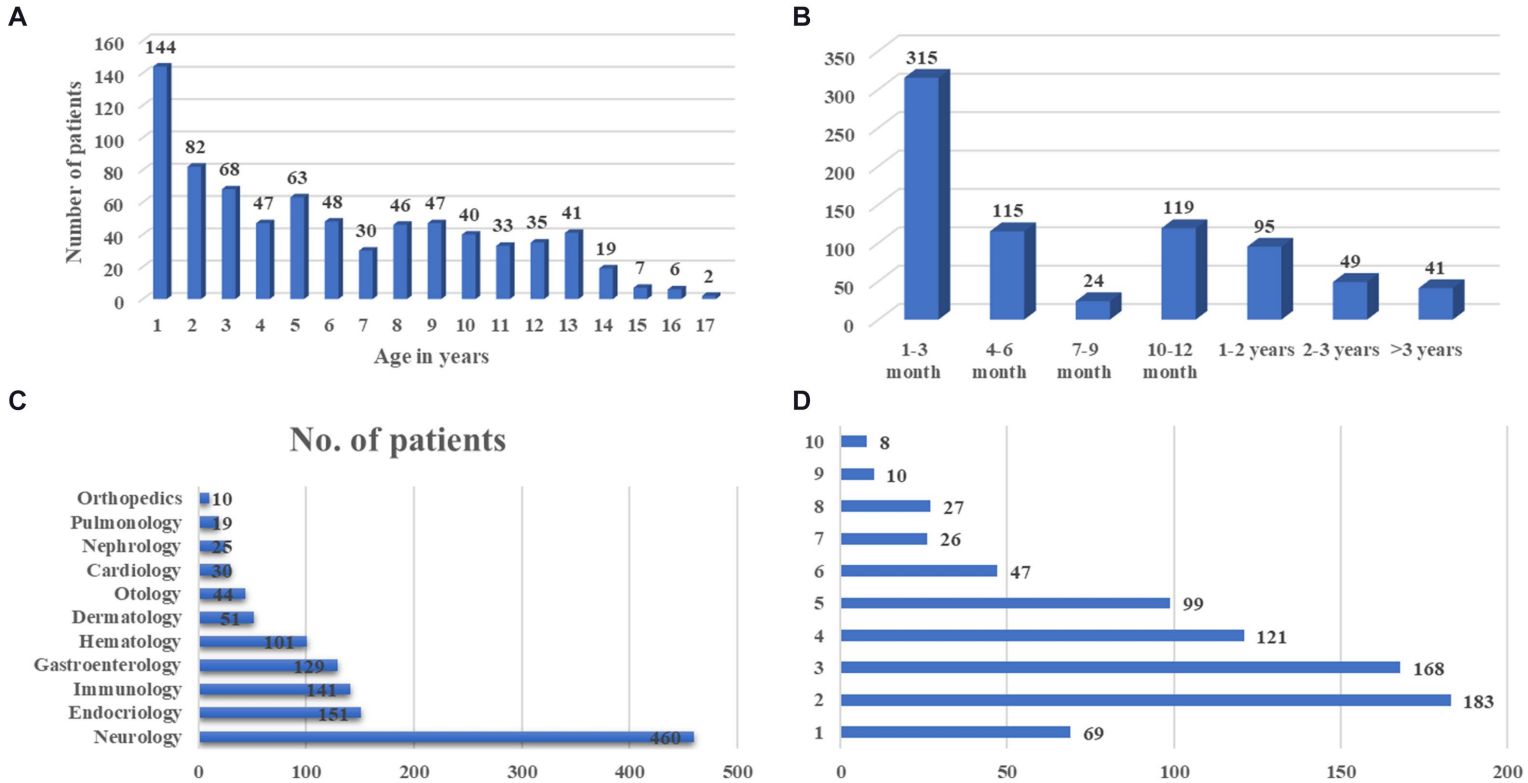
The basic clinical characteristics of UDP patients. **(A)** Age distribution of symptom onset at admission, **(B)** Course of disease at admission, **(C)** Distribution of organ involvement, **(D)** Number of presenting symptoms in each patient.

In our hospital, we have 758 cases with comprehensive UDP project data from January 2017 to December 2021. Table 1 and Figure 2 show demographic data for these 758 patients. Males made up 436 of them (57.5%). Moreover, half of the patients were under the age of 5, with only 15 patients over the age of 14 (Figure 2A). Average time from symptom onset to admission to UDP was 6.0 months (95% CI 10.5–12.6), and 430 patients (56.7%) sought medical attention within 6 months of seeing symptoms (Figure 2B). These patients visited an average of 1.8 clinics during their diagnostic journey. Except for 69 individuals (90.9%), all had more than one presenting symptom in various organs: 460 (60.7%) had neurology problems, 151 (19.9%) had endocrinology problems, and 141 (18.6%) had immunology problems (Figures 2C,D). We discovered that the clinical phenotypic, growth and development problem was the most common, accounting for 249 instances (32.9%), followed by nervous system abnormalities (220 cases, 29.0%). UDP has a diagnosis rate of 61.3%. Genetic testing was performed on 469 of the 758 patients, for a genetic diagnosis rate of 15.8%.

**Table 1:** Enrolled patient demographics.

	No (total *n* = 758)
Basic information	
Sex (male) (n, (%))	436 (57.5%)
Mean age of symptom onset (years)	6.0 ± 4.3
Mean months of before admitted to the UDP	6.0 (95%CI 10.5 ± 12.6)
Mean months to diagnosis after UDP admission	10.1 ± 8.7
Number of visited clinics before the project admission (n, (%))	
1	367 (48.4%)
2	230 (30.3%)
3	131 (17.3)
≥4	30 (4.0%)
Diagnosed number	465 (61.3%)
Genetic diagnosis	120 (15.8%)
Clinical phenotype (n, (%))	
Growth problem	249 (32.9%)
Nerve problem	220 (29.0%)
Rheumatic and immune problem	123 (16.2%)
Metabolic system	63 (8.3%)
Oncology and hematology problem	51 (6.7%)
Digestive problem	41 (5.4%)
Others	11 (1.5%)

We discovered that growth and development problems had the lowest confirmed rate of 113 (45.4%) and the longest mean duration before admission to the UDP, 14.7 ± 18.2 months, followed by the nervous system, 14.2 ± 14.5 months. The oncology and hematology system had the shortest duration, at 4.3 ± 7.9 months. The mean days to diagnosis following UDP admission for congenital multiple abnormality were the longest, 11.5 ± 12.9 days, and the shortest, 8.1 ± 4.5 days (Table 2). The metabolic diagnostic rate was the greatest, at 41.3%, while the rheumatic and immune system diagnosis rate was the highest, at 82.9%.

**Table 2:** Characteristics of different clinical phenotype.

	Sex (male) (%)	Age of symptom onset (years)	Time from symptom onset to admission to UDP (months)	Time to diagnosis after UDP admission (days)	Diagnosed number (%)	Genetic diagnosis (%)
Growth and development	130 (52.2)	5.8 ± 4.0	14.7 ± 18.2	11.5 ± 12.9	113 (45.4)	10 (4.0)
Nervous system	137 (62.3)	5.3 ± 4.0	14.2 ± 14.5	9.4 ± 6.8^a^	138 (62.7)	68 (30.9)
Rheumatic and immune system	68 (55.3)	6.5 ± 4.7	5.6 ± 7.8^a^^,^^b^	9.7 ± 6.9	102 (82.9)	13 (10.6)
Metabolic system	41 (65.1)	5.8 ± 4.5	10.8 ± 11.3^c^	11.3 ± 8.8	41 (65.1)	26 (41.3)
Oncology and hematology system	30 (58.8)	6.9 ± 4.4	4.3 ± 7.9^a^^,^^b^^,^^d^	10.1 ± 7.4	40 (78.4)	3 (5.9%)
Digestive system	22 (53.7)	7.6 ± 4.8	8.8 ± 11.3^a^^,^^b^	8.1 ± 4.5^a^	25 (61.0)	0 (0)
Others	8 (72.7)	6.6 ± 4.3	2.0 ± 1.8	6.0 ± 5.2	6 (54.4)	0 (0)

a Compared with the congenital multiple anomaly group, there is statistical significance, *p* < 0.05.

b Compared with the neurodevelopmental group, there is statistical significance, *p* < 0.05.

c Compared with the rheumatology and immunology group, there is statistical significance, *p* < 0.05.

d Compared with the metabolic group, there is statistical significance, *p* < 0.05.

Table 3 displays the genetic mutations discovered in this UDP project. The most commonly found gene mutations in UDP was ABCD1, IGHMBP2, ATM, CSF1R, SLC25A1 and ACSF3 (Table 4). Adrenal leukodystrophy (ALD) is a rare genetic metabolic disease caused by a mutation in the ABCD1 gene on the X chromosome, which results in the inability to metabolize extremely long chain fatty acids (VLCFA) normally, resulting in the gradual loss of white matter in the nervous system and adrenal cortex degeneration. The majority of patients with cerebral type (cALD) develop total paralysis or possibly death within a short amount of time. SMARD1 (autosomal recessive spinal muscular atrophy with respiratory distress) is a severe form of spinal muscular atrophy that damages the diaphragm and can cause respiratory distress. A recessive mutation in the IGHMBP2 gene causes this condition. The ataxia telangiectasia mutated gene is a critical gene in DNA damage testing. It was discovered in patients with telangiectatic ataxia, and about 1% of the population had heterozygotes with ataxia-telangiectasia mutated (ATM) deficiency. CSF1R gene linked leukoencephalopathy is a rare autosomal dominant inherited leukoencephalopathy characterized by increasing neuropsychiatric and motor symptoms. Imaging results include diffuse white matter degeneration, corpus callosum thinning, and brain tissue calcification. Primary axonal degeneration and myelin sheath loss are pathological features. Children with congenital myasthenia syndrome (CMS) caused by the SLC25A1 gene have an early onset, are generally intellectually impaired or delayed, and have metabolic problems. The R247Q and V49M genotypes are repeatable. Mutations in the ACSF3 gene cause methylmalonuria, a rare autosomal recessive condition characterized by an increase in methylmalonic acid (MA) and malonic acid (MMA) levels in the urine. This gene encodes an enzyme that is involved in the production of fatty acids.

**Table 3:** Genetic mutations discovered in UDP project.

Phenotype	Outcome
Neurodevelopmental	DEPCD5, CSF1R, PURA, CECE1, PEX12, SLC25A1, GFPT1, MPV17, KMT2B, POCR3A, PRRT2, SCN2A, ATP7A, TSEN2, SMN1, KCNQ2, IGHMBP2
Metabolic	GALC, NBAS, ADNP, UGT1A1, LMNA, GFM1, GPD1, ARG1, MTTP, SCL12A3, ACSFS
Rheumatology and Immunology	CD40LG, IFIH1, STAT1, ACVR1, ELANE
Congenital multiple anomaly	SECISBP2, RARB, LRBA, TBCK, FGFR3, FKBP14
Oncology and hematology diseases	PTPN11, ABCD1, PTPN11

**Table 4:** The most commonly found gene mutations in undiagnosed diseases program.

Gene	Location	NO. of cases	Disease
ABCD1	Xq28	7	congenital leukodystrophy
IGHMBP2	11q13.3	6	congenital myopathy
ATM	11q22.3	5	brain atrophy
CSF1R	5q32	5	neurodegenerative disease
SLC25A1	22q11.21	5	myasthenia gravis
ACSF3	16q24.3	5	short stature

After UDP discussion, 501 of the 758 patients that joined the UDP received preliminary diagnosis. After genetic and other laboratory tests, 36 of them were misdiagnosed. The success rate for diagnostics was 61.3%. There were 257 cases that were not confirmed by UDP discussion, and 27 were confirmed by genetic and other laboratory tests. In the context of UDP method evaluation, positive gene test is taken as the golden standard. Sensitivity indicates how likely an UDP method is to diagnose an unknown disease when patient has a positive gene test. Specificity refers to the ability of an UDP method to rule out the presence of a disease in someone who has a negative gene test. Positive predictive value (PPV) is the number of correct positive results of an UDP method by the total number of positive results. Negative predictive value (NPV) is the number of correct negative results an UDP method gives divided by the total number of negative results. As a result, the UDP method has a sensitivity of 94.5%, a specificity of 86.4%, a PPV of 92.8%, and an NPV of 89.5% (Table 5).

**Table 5:** Sensitivity and specificity of this procedure.

Procedure	Gold standard		Total
	+	−	
+	465	36	501
−	27	230	257
Total	492	266	758

Sensitivity: 94.5%; specificity: 86.4%; positive predictive value (PPV): 92.8%; negative predictive value (NPV): 89.5%.

### Illustrative cases

#### Ending the diagnostic odyssey

A six-year-old youngster was admitted to UDP. For the first 8 months, she had a neck lump and a fever. She was seen at two different children’s hospitals and had a battery of laboratory tests performed, including infection testing, rheumatology testing, and blood cancer testing. Despite being diagnosed with persistent Epstein–Barr virus infection, these extensive evaluations failed to establish the true cause. Additional results such as reductions in immunoglobulin G and M, CD4 and CD 8 cells were discovered after UDP admission and reevaluation at our hospital. The expert consortium suspected immunodeficiency disorder, such as primary immunodeficiency disease, and did gene analysis. As a confirmative diagnosis, compound heterozygous mutations in PIK3CD c.2869C > T, p.Arg957TrP were discovered.

For 3 years, a three-year-old girl was discovered to be anemic. She visited several hospitals and was diagnosed with iron deficiency anemia, but oral iron therapy was useless. The serum iron of the child was discovered to be lower after joining our hospital’s UDP, and it was suspected that the reason of iron shortage was a genetic condition. WES (whole exome sequencing) testing identifies iron-refractory iron deficiency anemia caused by a mutation in the TMPRSS6 gene.

#### Correction of an incorrect diagnosis

A 3-year-old girl was taken to UDP with increasing pericardial effusion and fever. Her clinical diagnosis had been infective endocarditis for 2 years prior to admission, despite no specific pathogen findings. We reanalyzed the patient’s clinical symptoms and laboratory testing after UDP admission. The presence of a skin rash, leukopenia, hemolytic anemia, proteinuria, blood ANA 1:1280 positive, anti-dsDNA 1:1000 positive, poor complement (C3 and C4), and serositis (recurrent pericardial effusion) matches the 2019 EULAR/ACR diagnostic criteria for systemic lupus erythematosus. The patient’s gene suggests a TNFAIP3 exons 7–8 heterozygous deletion.

## Discussion

As one of the core units of the National Children’s Medical Center, the children’s hospital of Fudan University focuses on dealing with difficult and severe illnesses, which is the hospital’s objective and obligation. Building and refining a diagnosis and treatment system for complicated and severe pediatric illnesses has become a critical way for the national team to achieve its goal. Given the rising international interest in undiagnosed children with RDs, we launched our UDP in January 2017 as a pilot project to assess the feasibility and effectiveness of a long-term UDP in China as well as a medical system for rare disease identification. As a result of the construction of a diagnosis and treatment system for difficult and critical diseases, the number of specialized outpatient clinics in the hospital has increased to 230, and 32 rare disease characteristic clinics have been established, which are listed in the national rare disease catalog. By creating the framework for clinical diagnosis and treatment of complicated and specialized illnesses, the hospital promotes specialized development. Our UDP diagnosis rate is 61.3%. Genetic testing was done on 469 of the 758 patients, yielding a 15.8% genetic diagnosis rate.

The UDP at the National Institutes of Health (NIH) has achieved the goal of making a comprehensive diagnosis for most patients, understanding of the disease pathogenesis, linking genetic and clinical findings and informing prognosis and therapy (7, 8). The Italian Undiagnosed Rare Diseases Network (IURDN) was established in 2016 as part of a bilateral relationship between Italy and the United States, which was backed by the Ministry of Foreign Affairs and International Cooperation in Italy. Over the course of 3 years, 71 cases were chosen and entered into the database (9, 10). In general, the UDP considers applicants in 8 to 12 weeks, with a 2 to 6 month waiting list for admission. The average number of patient visits per year was 130, with 40% of cases being pediatric and 60% being adult. On average, our UDP program admits 152 children per year, and all eligible children were admitted within 1 week. In Korea, the multigene panel accounted for 26.6% of the unexplained diseases program (2). The frequencies of other genetics diagnostic examinations ranged from 25 to 51.9% (11–13). Other UDPs had diagnostic success rates ranging from 35 to 38.9%, but ours is 61.3% (2, 14). Collaboration among multidisciplinary teams (MDT) can boost the cure rate of severe and critical illnesses dramatically (15). Our UDP team includes experts from rheumatology, immunology, hematology, cardiology, neurology, surgery, radiology, molecular diagnostics, and other fields. The consultation center is able to schedule consultations around the availability of the professional team.

According to certain studies, patients can wait 5–6 years for a molecular diagnosis after multiple clinic visits and 1–12 diagnostic tests (average 3–5) (16–18). Participants in our study visited an average of 1.8 clinics before to UDP admission. We also looked at medical accessibility, which was represented by the average time it took from the onset of symptoms to their first UDP visit, which was 6.0 months. We provided two paths to UDP admission: (1) a clinician referral letter and (2) a direct visit to the UDP central hospital for the patient. Our hospital has significantly increased the medical effectiveness of treating children with rare and undiagnosed diseases, enhanced the effectiveness of the consultation process, decreased the number of needless round trips for children, and provided better patient care through ongoing research and practice thanks to the assistance of the information-based treatment platform and multidisciplinary expert team.

There are still some limitations to this study. Firstly, not all children can afford genetic testing, resulting in some children being unable to identify diseases caused by genetic changes. Secondly, genetic testing cannot answer all rare diseases, and there are still some patients who have undergone multidisciplinary diagnosis and treatment, and those with negative genetic testing cannot find the cause. Finally, for all patients who have already been diagnosed, we still need long-term follow-up to understand the accuracy of diagnosis, the effectiveness of treatment, and the long-term survival of rare disease patients.

As stated by around 40% of parents of undiagnosed children (19, 20), misdiagnosis are well-known problems for people with RDs and their families, who are also tormented by stress and grief. Diagnostic lag is a critical issue that affects both patient and family quality of life (21, 22). We covered three instances of misdiagnosis prior to admittance to UDP in this essay. Clinicians should be aware of the numerous clinical presentations and new variants of a certain illness when making a diagnosis. Implementing nationwide UDP programs for unidentified patients based on MDT work mode and genetic technologies has the potential to greatly improve care quality.

## Data Availability

The raw data supporting the conclusions of this article will be made available by the authors, without undue reservation.
